# How the Proximal Pocket May Influence the Enantiospecificities of Chloroperoxidase-Catalyzed Epoxidations of Olefins

**DOI:** 10.3390/ijms17081297

**Published:** 2016-08-09

**Authors:** Alexander N. Morozov, David C. Chatfield

**Affiliations:** Department of Chemistry and Biochemistry, Florida International University, 11200 SW 8th St., Miami, FL 33199, USA

**Keywords:** heme-thiolate enzymes, chloroperoxidase, cytochrome P450, Compound I, proximal pocket, hydrogen bonding, helix dipole, catalytic reactivity, epoxidation, density functional theory

## Abstract

Chloroperoxidase-catalyzed enantiospecific epoxidations of olefins are of significant biotechnological interest. Typical enantiomeric excesses are in the range of 66%–97% and translate into free energy differences on the order of 1 kcal/mol. These differences are generally attributed to the effect of the distal pocket. In this paper, we show that the influence of the proximal pocket on the electron transfer mechanism in the rate-limiting event may be just as significant for a quantitatively accurate account of the experimentally-measured enantiospecificities.

## 1. Introduction

Chloroperoxidase (CPO), an enzyme secreted by the marine fungus *Caldariomyces fumago*, is a glycosylated heme-thiolate protein known for its exceptional versatility [1]. Along with its native function of halogenating organic substrates using chloride, bromide and iodide ions [2,3,4], CPO is also capable of many promiscuous activities, including peroxidase, catalase and cytochrome P450 (P450) types of reactions [1]. CPO-catalyzed reactions are of biotechnological and environmental importance [5], therefore attracting current research interest on both the experimental [6,7,8,9,10,11,12,13,14] and theoretical sides [15,16,17,18,19,20,21]. The catalytic cycle requires a two-electron oxidation of the ferric heme center, using hydrogen peroxide or other suitable peroxide, and the glutamic acid side chain (E_183_) in the distal pocket as a general acid-base catalyst, to form Compound I (CPO-I), a highly reactive oxyferryl porphyrin π-cation radical intermediate [15,20,22,23]. This first step is followed by the oxidation of a substrate, and the reaction cycle ends as CPO regains the native state.

The active center of CPO has both peroxidase and P450 features: like a peroxidase, CPO has a polar pocket on the distal side of the heme; like P450, CPO possesses a cysteine-derived thiolate axial ligand on the proximal side [24]. CPO’s distal pocket is connected to the protein surface by a roughly 10-Å wide channel, similar to P450s’ and with no analog in peroxidases [24,25]. In a previous computational study, we showed that CPO has the ability to mimic both peroxidase-like and P450-like distal pockets to tune the catalytic efficiency [18]. A key for the oxidative catalytic functions of CPO and P450s is the proximal thiolate ligand [24,26], which is a strong electron donor “pushing” electrons [27]. It took more than fifty years from the discovery of P450-type chemistry to establish that the “push” of the proximal thiolate results in a trade-off between the redox potential of the heme active center and the basicity of a distal axial ligand and, hence, constitutes a smart adaptation to the different stages of the reaction cycle [28,29], facilitating both O–O bond scission of a ferric hydroperoxy species [15,20,30] and subsequent oxygen transfer into a C=C double bond [31] or an inert C–H bond [28,29,32], as well as other oxygen transfer reactions [1,33].

**Figure 1:**
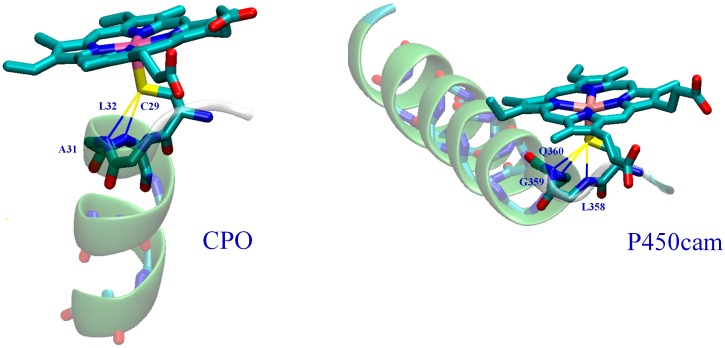
Proximal pockets of CPO and P450cam.

In heme-thiolate enzymes, the thiolate “push” is modulated by adjusting the thiolate-to-thiol character of the proximal ligand via sulfur/backbone-amide (NH–S) hydrogen bonds, a conserved feature of heme-thiolate proteins [34]. In CPO, the proximal thiolate ligand forms A_31_:NH–C_29_:S and L_32_:NH–C_29_:S hydrogen bonds (Figure 1) [24], made possible by the backbone conformation of the proximal pocket’s C–P–A–L peptide fragment [35]. P450cam contains the peptide fragment C–L–G–X, conserved in P450s, which forms L_358_:NH–C_357_:S, G_359_:NH–C_357_:S and Q_360_:NH–C_357_:S thiolate sulfur/backbone-amide hydrogen bonds via a similar backbone conformation [35]. Recently, we have summarized an extensive set of experimental data available on the effect of proximal NH-S hydrogen bonding in P450cam [21]. Briefly, there is accumulating evidence that NH-S hydrogen bonds play a chemical role: (1) by modulating the thiolate “push” effect through the alteration of π-electron donation by the proximal thiolate [36]; (2) by modulating the heme reduction potential by up to ~200 mV per NH–S hydrogen bond [36,37,38]. NH–S hydrogen bonds also play a structural role, in protecting and stabilizing Fe-S coordination [39]. On the theoretical side, calculations of the effect of proximal NH–S hydrogen bonds on oxidations catalyzed by a model heme-thiolate Compound I, using (SH)^−^ to represent the proximal ligand, indicate that NH–S bonding has an influence on the chemoselectivity of Compound I toward hydroxylation vs. epoxidation [40,41]. Theoretical simulations of proximal pocket/sulfur hydrogen bonding in nitric oxide synthase showed that such bonding may affect the distribution of the thiolate/porphyrin unpaired spin [42]. The effect of hydrogen bonding to the distal oxygen of model manganese(IV)-oxo and iron(IV)-oxo oxidants on the oxidants’ ability for oxygen atom transfer was studied [43]. However, to the best of our knowledge, there are no published theoretical results regarding the possibility that the proximal helix and the NH–S bonds may affect the enantiospecificity of oxidations catalyzed by heme-thiolate enzymes. In addition to NH–S hydrogen bonding, the electropositivity of the N-terminus of the proximal α-helix also modulates the anionic character of the proximal thiolate ligand in CPO and P450 [34]. The helix dipole moment is in the range of 3.5–5 D per peptide residue, according to both experiment and theoretical calculation [44,45]. Elements of secondary structure are known to play mechanistic roles in folding and post-translational modification of heme-containing proteins [46,47,48,49]. The electric field at helix termini is strong enough to influence protein folding, ligand binding and enzymatic reactions [44,50]. Experimental studies of heme-thiolate model complexes showed that adding the proximal helix to a model compound increases the Fe^III/II^ redox potential by 130 mV for a CPO model and by 70 mV for a P450cam model [51]. In view of this, it is not surprising that the proximal helix may amplify the NH–S effect in heme-thiolate enzymes, as experiments suggest [51].

**Figure 2:**
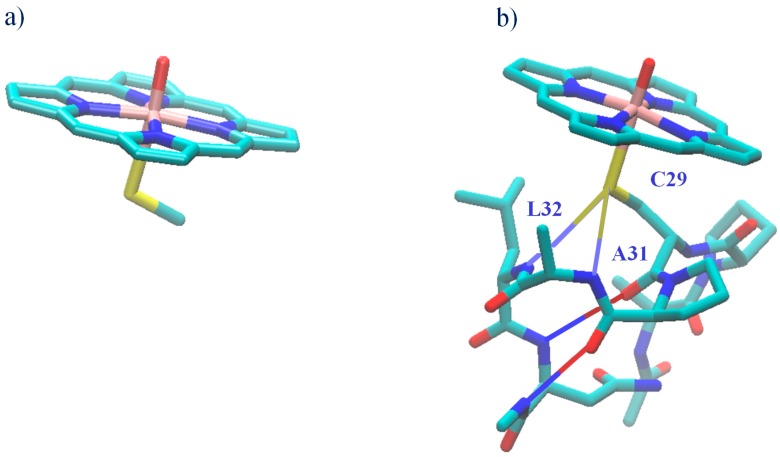
Bare-thiolate, CPO-I-A (**a**); and CPO-like, CPO-I-B (**b**) proximal pocket models of CPO-I.

CPO’s ability to catalyze P450-type enantiospecific epoxidations of olefin substrates [52,53] is of particular interest, as chiral epoxides are synthons for various pharmaceutical and industrial applications. The experimental data described above suggest that the heme thiolate’s secondary coordination sphere and the proximal helix are of importance for epoxidations catalyzed by heme-thiolate enzymes. Our recent DFT studies provide theoretical evidence that the combined effect of the proximal NH–S bonds and the dipole moment of the proximal helix significantly enhances CPO’s reactivity toward the epoxidation of olefinic substrates [21]. When the environment of the proximal thiolate ligand (Figure 2B) is included in the model, the rate limiting barrier for C–O bond formation on the doublet spin surface for CPO-catalyzed epoxidation of *cis-β*-methylstyrene (CBMS) to form the 1S2R enantiomer is lowered by ~4.6 kcal/mol relative to the ~15 kcal/mol barrier for the bare-thiolate model (Figure 2A). In this nomenclature, the alpha carbon is labeled 1 and followed by its chirality, here S, while the beta carbon is labeled 2 and followed by its chirality, here R. It was estimated that the dipole moment of the proximal helix contributes ~1/3 of the decrease [21]. For the 1S2R enantiomer on the doublet spin surface, we found that the effects of CPO’s proximal pocket change the preferred electron transfer mechanism. For the bare thiolate model of CPO-I, the first and rate-limiting event is the reduction of Fe^IV^ to Fe^III^ by an electron transferred from the C=C π bond to the oxyferryl π* orbital. With the proximal pocket added to the model, though, the reduction of the porphyrin moiety via electron transfer to the a_2u_+σ_S_ orbital is the first and rate-limiting event [21]. A detailed description of the orbitals important for the reactivity of heme thiolate enzymes can be found elsewhere [21,54].

The study of the 1S2R reaction pathway described above [21] clearly indicates that the effect of the secondary coordination sphere and proximal helix on the electron donating properties of the proximal thiolate is anisotropic in nature and hence may interfere differently with different chiral transition states. This observation provides a motivation for asking a rather unconventional question: Is it possible that the proximal environment may also have a significant effect on the enantiospecificity of a heme-thiolate-catalyzed epoxidation reaction? Herein, we address this question by comparing the combined effect of NH–S hydrogen bonding and the proximal helix’s dipole moment on the rate-limiting barrier for the 1R2S doublet pathway of the CPO-catalyzed epoxidation of CBMS with the result for the 1S2R pathway obtained previously. CPO-I converts CBMS into the epoxide with 96% enantiomeric excess of the 1S2R over the 1R2S product [52], which corresponds to a free energy difference of ~2.0 kcal/mol in favor of the 1S2R enantiomer at 300 K. This underscores that significant levels of enantiospecificity can result from small free energy differences, making it difficult to identify the underlying causes of enantiospecificity with certainty. It is generally assumed that the distal binding site controls enantiospecificity by providing steric or specific interactions. In this paper, we shall show that in the case of CPO-catalyzed epoxidation of CBMS, the effect of the proximal pocket on the enantiospecificity is of the same order of magnitude (~1.0 kcal/mol) as that of the distal pocket. Thus, it is possible that the effect of the proximal environment on the enantiospecificity of a heme-thiolate catalyzed epoxidation reaction may be significant. The underlying reason for this is that the secondary coordination sphere of the proximal thiolate and the dipole of the proximal helix determine the preferred electron transfer mechanism for the rate-limiting event. We found that in the case of CPO-I catalyzed epoxidation of CBMS, electron transfer to different oxyferryl π* orbitals results in the energy splitting of the transition states leading to the 1R2S and 1S2R product epoxides. This difference was not observed when the heme thiolate’s secondary coordination sphere and the proximal helix were included in the model. In this case, the proximal NH–S bonds and helix dipole reduce the electron donating properties of the proximal thiolate. This reduction in electron donation, in effect an electron withdrawal, is greater for π than for σ orbitals and results in the electron transfer being to the a_2u_+σ_S_ rather than to a π* orbital. Due to this change in the electron transfer mechanism, the 1R2S and 1S2R prechiral transition states become degenerate. We conclude that for heme-thiolate enzymes, the effect of the secondary coordination sphere and of the proximal helix on the electron-donating properties of the proximal thiolate ligand has to be included to provide quantitatively reliable energy differences for prechiral transition states.

Our work employs model systems, described in the Computational Methods section, chosen to isolate the influence of the heme thiolate’s secondary coordination sphere and the proximal helix on CPO-I catalyzed epoxidation of CBMS. Thus, the model systems do not explicitly represent the distal binding pocket. We note that a large amount of conformational sampling is needed to quantify the influence of protein-CBMS steric interactions directly within meaningful margins of error. This is because steric interactions can fluctuate substantially relative to the small energy differences causing the enantiomeric excess. Our model-system approach allows us to focus on the influence of the proximal region without the conformational sampling issue.

## 2. Computational Details

### 2.1. Methods

Unrestricted DFT calculations of the doublet spin surfaces were carried out without symmetry restrictions using the B3LYP [55,56] hybrid density functional (UB3LYP) with the LANL2DZ effective core potential (ECP) double-ζ basis set for Fe [57] and the 6-31G* basis set for H, C, N, O [58] and S [59] atoms (basis set B0) using NWChem 6.1 software [60]. The stability of the density functions obtained was checked with the B0 basis set using Gaussian-09 [61]. The LANL2TZ + ECP triple-ζ basis set for Fe [62] and the 6-311++G** basis set for H, C, N, O [63] and S [64] atoms (basis set B1) were used for energy refinement. Frequency calculations with basis set B0 were used to obtain zero point energy (ZPE) corrections, which are included in the energies of all stationary points given. Natural population analysis [65] (NPA) of spin/charge densities was carried out using NBO 6.0 software [66].

### 2.2. CPO-I Models

CPO-I was modeled as an R^−^-Fe^4+^O^2−^(N_4_C_20_H_12_)^−^ species in which the heme moiety lacks the vinyl and propionate side chains. This 43-atom model of CPO-I, CPO-I-A, employed R^−^ = (SCH_3_)^−^ (Figure 2) and constitutes the thiolate model, which lacks the axial sulfur’s secondary coordination sphere and the proximal helix. The simulations of CPO-I-A were performed without geometric constraints. All transition state structures have one imaginary normal mode frequency, while all stable structures have only positive, real frequencies.

The CPO-I model used to study the proximal pocket effect (CPO-I-B) employed the R^−^ = CH_3_–NH–Asn–Leu–Ala–Pro–Cys^−^–Pro–Ala–CO–CH_3_ peptide fragment (Figure 2B), which includes the proximal amino acid residues having C_α_ atoms within 8 Å of the proximal sulfur. Model B consists of 141 atoms and includes the secondary coordination sphere of the axial sulfur provided by the A_31_:NH-C_29_:S and L_32_:NH-C_29_:S hydrogen bonds and the immediate steric environment of the axial sulfur, as well as the proximal α-helix. The proximal α-helix was included so that the dipole moment deriving from the C_29_:CO–N_33_:NH and P_30_:CO–A_34_:NH backbone hydrogen bonds is represented in the model. The full proximal α-helix of CPO has 3 more hydrogen bonds formed by the backbone of residues 35 to 38. These residues were not included due to computer resource limitations. Thus, the effect of the proximal helix dipole in our Model System B represents a lower bound to the actual effect. The proximal peptide fragment of CPO-I-B was constrained to maintain backbone and side-chain hydrogen bonds, backbone ϕ,*ψ* dihedrals and the orientation of the proximal helix relative to the heme moiety as in the crystal structure of CPO (PDB Code 1CPO) [24]. The stationary points of the CBMS/CPO-I-B reactant and transition state complexes have extra normal modes with imaginary frequencies caused by these constraints. The NH–S hydrogen bonds were not constrained. The details of the constraints applied are given in the Supplementary Materials (SM) (Data S1).

### 2.3. Initial Structures for 1R2S and 1S2R CPO-I/CBMS Transition State Complexes

For both Models A and B, the C_4_ rotational symmetry of the porphyrin moiety results in four CPO-I/CBMS transition state complex conformations that are nearly degenerate in energy. In our previous work, we developed a molecular mechanics (MM) parameterization of the transition state using the quantum mechanics to molecular mechanics (Q2MM) method [67] and used it to dock the low spin CPO-I/CBMS transition state complex into the apoenzyme scaffold of CPO [18]. It was found that three of the four possible 1S2R CPO-I/CBMS transition state conformations were unrealistic because of significant steric overlap between CBMS and the distal pocket residues [18]. In this work, we used the same method to carry out the docking of four possible 1R2S CPO-I/CBMS low spin prechiral transition state conformations and found that, as for the 1S2R pathway, only one conformation is allowed by the steric restrictions of the distal pocket (Figure S1). The Cartesian coordinates of the 1R2S and 1S2R docked structures are given in the SM (Data S2A). The pertinent portions of these structures were used to initiate the fully quantum mechanical (QM) transition state searches on Model Systems A and B. The Cartesian coordinates of all stationary points are given in the SM (Data S2B).

## 3. Results

**Figure 3:**
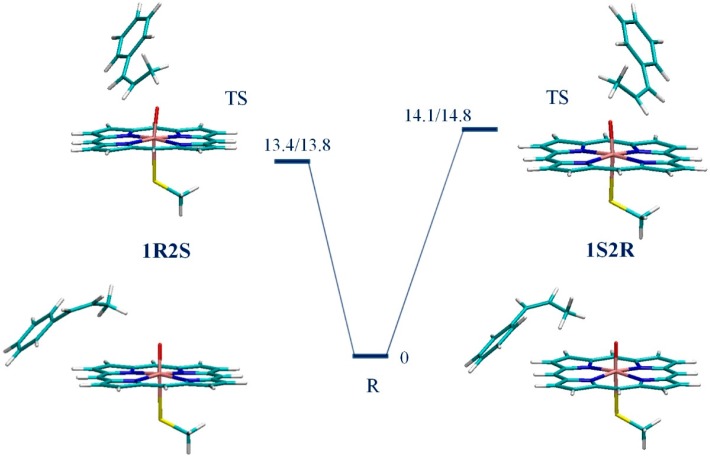
The UB3LYP/B1//B0 potential energy surfaces (in kcal/mol) connecting the reactant states R and the rate-limiting transition states **TS** leading to the formation of a C_β_–O bond on the doublet potential energy surfaces for epoxidation of *cis-β*-methylstyrene (CBMS) by CPO-I-A to give 1R2S and 1S2R products.

**Figure 4:**
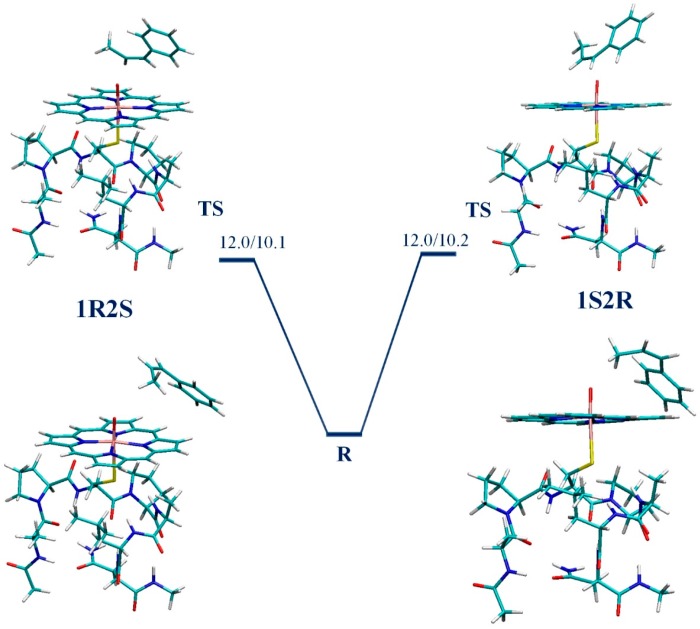
The UB3LYP/B1//B0 potential energy surfaces (in kcal/mol) connecting the reactant states **R** and the rate-limiting transition states **TS** leading to the formation of a C_β_–O bond on the doublet potential energy surfaces for 1R2S and 1S2R epoxidation of CBMS by CPO-I-B.

We have shown previously that, on the doublet surface, the epoxidation reaction takes place in two steps. The first and rate-limiting step is the formation of the C_β_–O bond, which is followed by the facile formation of the C_β_–O bond and ring closure [18]. The UB3LYP/B1//B0 potential energy surfaces (PES) connecting the reactant state (**R**) and the rate-limiting transition state (**TS**) leading to the formation of a C_β_–O bond were calculated for the doublet 1R2S pathway of CPO-I-A and CPO-I-B catalyzed epoxidation of CBMS. The transition states were found by scanning the C_β_–O distance followed by saddle-point optimization. The reactant states were found by optimizing along the transition mode of the **TS** in the direction of increasing C_β_–O distance. The results calculated here for the 1R2S pathway were compared to those for 1S2R from our previous work [21]. For both Models A and B of CPO-I, the reactant states leading to the 1R2S and 1S2R products are CPO-I + CBMS bound complexes degenerate in energy and characterized by the weak interaction of the methyl group of CBMS and the oxygen of CPO-I; the transition states are the results of the oxyferryl radical attack on the C=C double bond of the substrate (Figure 3 and Figure 4). The natural spin densities and charges (Table 1) show that the 1R2S and 1S2R electron transfer mechanisms are identical. Furthermore, the 1R2S spin densities and charges confirm our previous result for the 1S2R reaction [21], namely that for the CPO-I-B model the rate-limiting kinetic event is an electron transfer from the C=C π bond to the a_2u_+σ_S_ thiolate-porphyrin orbital, while for the bare-thiolate CPO-I-A model, the electron is transferred to one of the oxyferryl π* orbitals. The important bond lengths of the stationary points are given in Table 1. The PESs and optimized structures for CPO-I-A and CPO-I-B catalyzed epoxidation of CBMS are shown in Figure 3 and Figure 4, respectively. The PESs include ZPE corrections calculated at the UB3LYP/B0 level. For the bare-thiolate model, CPO-I-A, the calculations show that the 1R2S reaction pathway is favored by 1 kcal/mol (Figure 3). For CPO-I-B, the calculations show that the proximal pocket lowers the rate-limiting **TS** barrier by 3.7 kcal/mol on the doublet spin 1R2S surface, as compared to 4.6 kcal/mol calculated previously [21] for the doublet spin 1S2R surface. The influence of the proximal pocket thus removes the relative favorability that the 1R2S reaction pathway **TS** has in the absence of the apoprotein (Figure 4).

## 4. Discussion

The experimentally-measured enantiomeric excesses of CPO-catalyzed epoxidations of various alkenes vary in the range 66%–97% [52]. This translates into free energy differences of ~0–3 kcal/mol at room temperature, which is a small number compared to the thermal fluctuations of a typical ~20,000-atom molecular model of the solvated CPO/substrate complex in an NPT simulation (number of atoms, pressure, and temperature constant), leading to the difficulty of accurate sampling. A comprehensive study of a stereoselective reaction should produce an accurate free energy potential surface that includes appropriate conformational averaging and connects substrate/enzyme, transition-state/enzyme and product/enzyme complexes. With current computational means, a molecular dynamics (MD) trajectory of ~100 ns–~1 μs is achievable if a classical MM force field is employed, allowing the testing of the binding hypothesis, i.e., the notion that the favorability of substrate binding conformations parallels the enantiomeric excess of the epoxide product [68]. Previously, we carried out an extensive MM simulation to distinguish binding potential wells of the CPO/CBMS complex from which the reaction to 1S2R and 1R2S epoxide products may occur; the flatness of the calculated free energy landscape rules out the binding hypothesis [17]. This is because CBMS floats relatively freely in the active site, as it is oriented by nonpolar interactions. It follows that the reaction is under kinetic control, and QM/MM transition state calculations are needed to identify the source of stereoselectivity. This presents a computational challenge, foremost because a substantial amount of conformational sampling is likely to be required to adequately reproduce the net influence of the distal region. For this purpose, a Q2MM model might be sufficient. However, as the present study shows, for heme-thiolate enzymes, an explicitly QM level of theory is required to account for the effect of the proximal region on the electron transfer mechanism.

**Figure 5:**
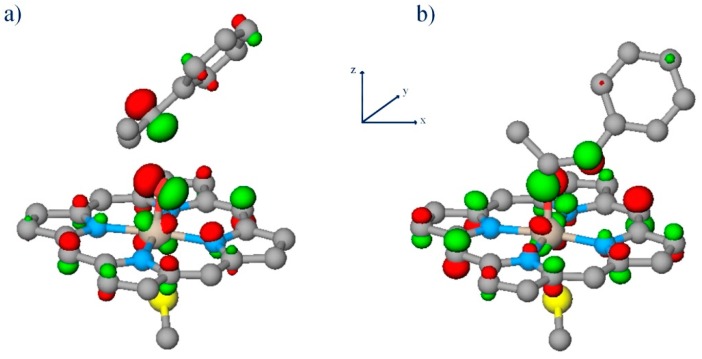
Oxyferryl π* attack on C=C bond for model CPO-I-A: (**a**) LUMO in β manifold of 1R2S **TS**; (**b**) LUMO in β manifold of 1S2R **TS**.

The calculations presented here for CPO-I-A catalyzed epoxidation of CBMS on the doublet spin surface show that for the bare-thiolate model without the influence of the proximal pocket, CPO-I favors the formation of the 1R2S over the 1S2R enantiomer by 1 kcal/mol. Since the bound complexes were found to be degenerate in energy, the transition states are the key to understanding this phenomenon. On the 1R2S pathway, the C=C π bond is attacked by the oxyferryl π_xz_* electron, while on the 1S2R pathway, the attack is by the π_yz_* electron (Figure 5). This stereo difference is the result of the restrictions imposed by the distal pocket on the possible orientation of the substrate in the 1R2S and 1S2R transition states with respect to the π_xz_* and π_yz_* orbitals (Figure 5). The electron transfer to the different π* orbitals results in the energy splitting of the 1R2S and 1S2R transition states because of uneven π-donation by the proximal thiolate ligand to these orbitals. When the proximal NH-S bonds and helix dipole are included by means of the CPO-I-B model, there is a larger withdrawing effect on π-donation than on σ-donation by the proximal thiolate. As a result, the electron from the C=C π bond is transferred to the a_2u_+σ_S_ thiolate-porphyrin orbital, rather than to a π* orbital, for both the 1R2S and 1S2R channels. Hence, one would expect the chiral transition states to be degenerate, as we have found with the calculations presented.

## 5. Conclusions

For heme-thiolate enzymes, contrary to the general understanding, the proximal region can have nearly as great an effect on the enantiospecificity of the epoxidation reaction as the distal region. This is possible because the proximal pocket determines the preferred electron transfer mechanism, which is either to the a_2u_+σ_S_ orbital or to one of the π* orbitals. The latter case allows an interplay of the substrate orientation in the distal pocket with the asymmetry of π-donation by the proximal thiolate. In the case of CPO, the calculated 1 kcal/mol difference in favor of the 1R2S enantiomer for the bare-thiolate (CPO-I-A) catalyzed epoxidation of CBMS on the low spin surface results in a difference of ~3 kcal/mol with respect to the experimental value of 1.96 kcal/mol in favor of the 1S2R epoxide product, determined from the enantiomeric excess [52]. The experimental value reflects the total effect of the enzyme in favor of the 1S2R channel. The results for CPO-I-B catalyzed epoxidation of CBMS show that ~1 kcal/mol out of the net ~3 kcal/mol difference is due to the combined influence of the proximal thiolate’s secondary coordination sphere (especially NH–S hydrogen bonding) and the proximal helix dipole. The inclusion of these factors reproduces the correct electron transfer mechanism. The remaining ~2 kcal/mol presumably reflects the net influence of the distal pocket. Since the expected effect of the distal and proximal pockets of CPO are of the same order of magnitude, we conclude that proper modeling of the proximal pocket is necessary to correctly calculate the enantiospecificities of heme-thiolate-catalyzed epoxidations.

## Figures and Tables

**Table 1:** Natural group spin densities/charges and bond lengths (Å) of the optimized structures on the doublet spin potential energy surfaces (PES).

		Natural Spin Densities/Natural Atomic Charges								Bond Lengths		
		S–R	Por	Fe	O	C_β_H	C_α_H	R_1_^+^	R_2_^+^	S–Fe	Fe–O	O–C_β_
1S2R												
**R**	A	−0.75/−0.05	−0.30/−0.49	1.10/0.91	0.95/−0.37	0.00/0.06	0.00/−0.03	0.00/−0.05	0.00/0.02	2.619	1.623	–
	B	−0.60/−0.27	−0.50/−0.32	1.15/0.95	0.95/−0.36	0.00/0.06	0.00/−0.03	0.00/−0.05	0.00/0.02	2.776	1.619	–
**TS**	A	−0.70/−0.08	−0.30/−0.58	0.95/0.90	0.75/−0.44	−0.10/0.15	0.30/0.04	0.10/−0.02	0.00/0.03	2.554	1.705	1.985
	B	−0.30/−0.40	−0.25/−0.51	1.40/0.96	0.50/−0.42	−0.05/0.18	−0.20/0.08	−0.10/0.07	0.00/0.04	2.570	1.658	2.099
1R2S												
**R**	A	−0.76/−0.05	−0.30/−0.49	1.10/0.91	0.96/−0.37	0.00/0.06	0.00/−0.03	0.00/−0.05	0.00/0.02	2.624	1.623	–
	B	−0.59/−0.27	−0.48/−0.32	1.15/0.95	0.95/−0.36	0.00/0.06	0.00/−0.03	0.00/−0.05	0.00/0.02	2.776	1.619	–
**TS**	A	−0.65/−0.08	−0.28/−0.59	0.91/0.89	0.76/−0.43	−0.11/0.15	0.27/0.05	0.10/−0.01	0.00/0.03	2.512	1.702	1.996
	B	−0.30/−0.41	−0.26/−0.50	1.38/0.96	0.52/−0.42	−0.06/0.18	−0.17/0.08	−0.11/0.07	0.00/0.04	2.576	1.658	2.116

S-R: proximal sulfur together with rest of R^—^ moiety (SCH_3_ for model A; sulfur with proximal helix for model B); Por: porphyrin; R_1_^+^: benzylic group of CBMS; R_2_^+^: methyl group of CBMS.

## Supplementary Materials

Supplementary materials can be found at www.mdpi.com/1422-0067/17/8/1297/s1.
