# Supplementary material for: How the Proximal Pocket May Influence the Enantiospecificities of Chloroperoxidase-Catalyzed Epoxidations of Olefins
Source: Int J Mol Sci. 2016 Aug 9;17(8):1297. doi: 10.3390/ijms17081297 (PMC5000694; doi:10.3390/ijms17081297)
Supplement: Supplementary file 1 [file ijms-17-01297-s001.pdf]

Supplementary Materials: How the Proximal Pocket May Influence Enantiospecificities of Chloroperoxidase-Catalyzed Epoxidations of Olefins

Alexander N. Morozov and David C. Chatfield

Table of Contents

Figure S1: Steric overlaps in four possible CPO-I/CBMS transition state complexes S1
Data S1: Constraints on the proximal peptide fragments of model B S2
Data S2A: Cartesian coordinates of the 1R2S and 1S2R CPO-I/CBMS transition state docked structures S3
Data S2B: Cartesian coordinates of the 1R2S stationary points S10

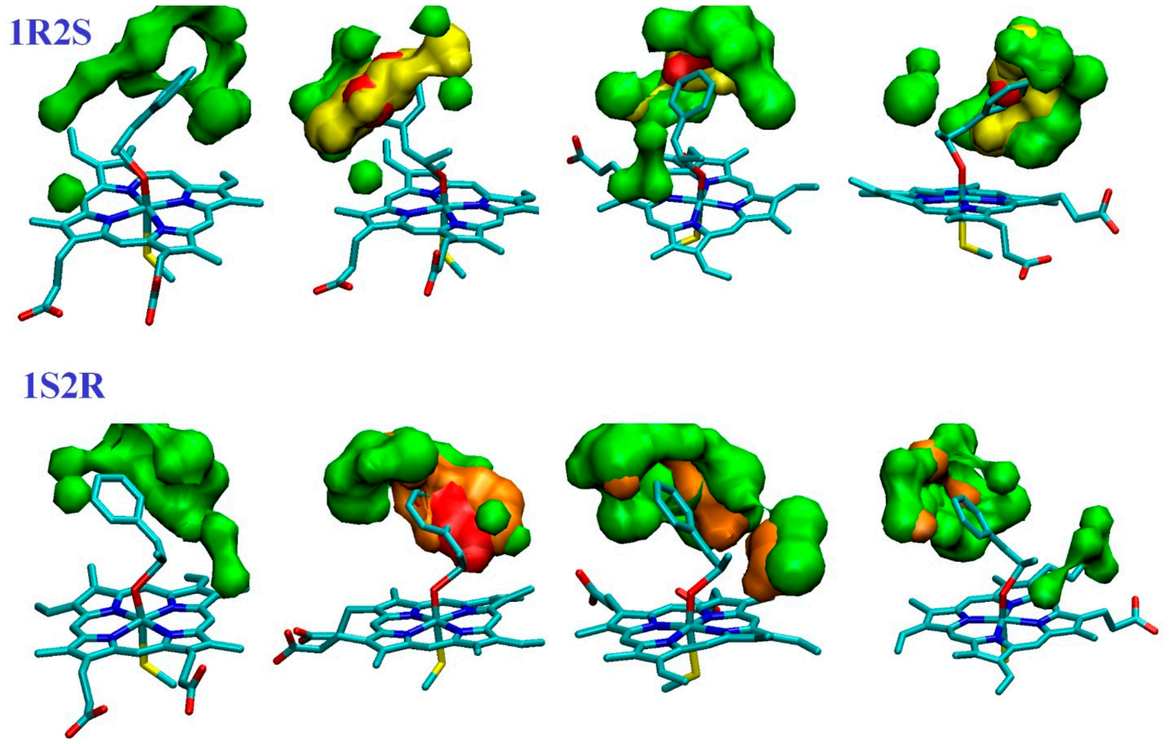

Figure S1. Steric overlaps in four possible CPO-I/CBMS transition state complexes. Color code: green–atoms within 3 Å of the substrate; yellow-atoms within 2 Å; red–atoms within 1 Å.

Data S1. Constraints on the proximal peptide fragments of model B. Geometry constraints on the proximal peptide fragment that were applied to maintain backbone and side-chain hydrogen bonds, backbone ϕ,ψ dihedrals, and the orientation of the proximal helix relative to the heme moiety as in the crystal structure 1CPO.

```
geometry adjust noautosym nocenter
 zcoord
#prox helix/heme orientation
 torsion 23 21 58 59  constant
 torsion 21 58 59 63  constant
 torsion 58 59 63 64  constant
 torsion 59 63 64 65  constant
#backbone φ,ψ
 torsion 64 65 66 67  constant
 torsion 65 66 67 68  constant
 torsion 67 68 69 70  constant
 torsion 68 69 70 71  constant
 torsion 70 71 72 73  constant
 torsion 71 72 73 74  constant
 torsion 73 74 75 76  constant
 torsion 74 75 76 77  constant
 torsion 65 64 63 78  constant
 torsion 64 63 78 79  constant
 torsion 78 79 80 81  constant
 torsion 79 80 81 82  constant
 torsion 81 82 83 84  constant
 torsion 82 83 84 85  constant
#backbone hb
 bond 74 110 constant
 bond 77 121 constant
#prox helix ASN side chain -loop backbone hb
 bond 84 150 constant
 bond 97 160 constant
end
end
```

Data S2A. Cartesian coordinates of the 1R2S and 1S2R CPO-I/CBMS transition state docked structures.

1R2S

C	-0.481711	4.417583	6.186582
H	-0.209224	5.230765	6.854150
C	0.005338	3.126925	6.412628
H	0.656764	2.935898	7.261371
C	-1.325936	4.650680	5.097429
H	-1.712814	5.649330	4.912431
C	-0.338079	2.077833	5.564395
H	0.042705	1.087870	5.775651
C	-1.673006	3.605527	4.248117
H	-2.326205	3.792047	3.399748
C	-1.186331	2.289107	4.450249
C	-1.620705	1.269662	3.516189
H	-2.356052	1.620697	2.796679
C	-1.200440	-0.038444	3.319987
H	-1.823671	-0.617552	2.646743
C	-0.318128	-0.857808	4.224427
H	-0.108679	-1.824130	3.758784
H	0.641613	-0.364921	4.401332
H	-0.802242	-1.040626	5.193855
O	0.178511	0.217053	1.899009
Fe	0.008332	0.177802	0.220112
N	-1.439725	-1.193588	0.073482
N	1.404487	-1.281143	0.132845
N	1.488820	1.559653	0.089914
N	-1.366574	1.629969	0.084663
C	1.173384	-2.631395	0.221306
C	2.424084	-3.351392	0.271473
C	3.415222	-2.419988	0.206501
C	2.769044	-1.131162	0.126920
C	2.842997	1.330755	0.063172
C	3.559527	2.585474	0.079971
C	2.622453	3.571449	0.137445
C	1.332203	2.920252	0.144485
C	-1.134356	2.983298	0.141365
C	-2.380784	3.706714	0.091759
C	-3.371024	2.777357	-0.017374
C	-2.727777	1.486954	-0.020536
C	-2.792141	-0.973712	-0.056123
C	-3.504031	-2.227395	-0.091072
C	-2.570927	-3.211129	0.030131
C	-1.287878	-2.559066	0.124749
C	-0.077252	-3.230377	0.222409
H	-0.111772	-4.314877	0.269607
C	3.443896	0.081696	0.068002
H	4.529463	0.048676	0.059034
C	0.115719	3.586021	0.185480
H	0.142975	4.670988	0.224995

C	-3.396389	0.273690	-0.107424
H	-4.477688	0.303206	-0.204397
H	4.487065	-2.572343	0.212558
H	2.512899	-4.428392	0.337219
H	2.771005	4.643205	0.168676
H	4.637846	2.678661	0.057749
H	-2.468712	4.785549	0.115906
H	-4.439365	2.935175	-0.095132
H	-4.577590	-2.324157	-0.192397
H	-2.719330	-4.283501	0.044441
S	0.067067	0.462846	-2.366794
C	0.955726	-0.764252	-3.166226
H	1.683709	-0.436409	-3.913828
H	1.632141	-1.214948	-2.419900
H	-0.055781	-2.684509	-3.526039
C	0.147674	-1.812937	-3.952935
C	-0.837776	-1.107667	-4.870796
N	-2.149053	-1.312122	-4.652035
C	-3.209636	-0.690644	-5.453855
C	-3.168512	0.848097	-5.408666
N	-2.819143	1.426663	-4.257784
C	-2.759503	2.887019	-4.124999
C	-1.615656	3.515573	-4.942790
N	-0.410462	2.969226	-4.800183
C	0.745017	3.482501	-5.539759
C	0.554660	3.357897	-7.062102
N	0.022723	2.222212	-7.510835
C	-0.214760	2.018198	-8.935015
C	-1.230056	3.008369	-9.492561
N	-2.272511	3.316287	-8.728149
N	1.071678	-2.654126	-4.693758
C	1.206779	-3.954252	-4.447163
C	2.267692	-4.696630	-5.287697
N	3.267536	-3.724258	-5.751426
C	3.094404	-3.033737	-6.900173
C	4.185359	-2.031301	-7.273891
N	4.028335	-1.412964	-8.593088
C	4.844921	-1.674738	-9.608399
C	4.597387	-0.908986	-10.906471
H	4.470495	0.124470	-10.620858
H	3.723438	-1.259825	-11.435172
H	5.479569	-0.976685	-11.525802
O	5.784568	-2.460687	-9.525502
H	3.265748	-0.777767	-8.685250
H	5.103443	-2.599472	-7.266273
C	4.222376	-0.924942	-6.225337
H	5.269554	-0.588522	-6.067446
H	3.617893	-0.056260	-6.565179
H	3.800926	-1.297516	-5.267156
O	2.125486	-3.231162	-7.644981
C	4.539884	-3.891652	-5.018282
H	5.278958	-4.429254	-5.648041

H	4.944235	-2.902273	-4.720367
C	4.123883	-4.714927	-3.813960
H	4.982679	-5.275727	-3.386652
H	3.677972	-4.079164	-3.019895
C	3.099934	-5.645947	-4.420293
H	3.583985	-6.435832	-5.033239
H	2.478016	-6.129088	-3.636793
H	1.775099	-5.162999	-6.126040
O	0.500608	-4.552873	-3.634826
H	1.602435	-2.196372	-5.402617
O	-0.432093	-0.358614	-5.755098
C	-2.718887	-2.251823	-3.670984
H	-2.097537	-3.168950	-3.607203
H	-2.792395	-1.772194	-2.672368
C	-4.079514	-2.532781	-4.261912
H	-4.018160	-3.295455	-5.066656
H	-4.789145	-2.886468	-3.484365
C	-4.490350	-1.221337	-4.801086
H	-4.836834	-0.545338	-3.990603
H	-5.304401	-1.331991	-5.548191
H	-3.094276	-0.945566	-6.495698
O	-3.454101	1.503384	-6.410744
H	-2.585042	0.898838	-3.444681
H	-3.656641	3.300157	-4.561282
C	-2.651652	3.286259	-2.658564
H	-3.429828	2.760618	-2.065111
H	-1.652645	3.007878	-2.259280
H	-2.781283	4.385197	-2.555115
O	-1.839523	4.451897	-5.731673
H	-0.241307	2.196852	-4.192822
H	0.820860	4.543054	-5.350879
C	2.024716	2.769202	-5.093594
H	1.861933	1.676350	-5.198899
H	2.844654	3.079685	-5.775266
C	2.478233	3.042562	-3.652533
H	1.617816	2.906545	-2.962369
C	2.914891	4.492524	-3.487035
H	3.232396	4.674346	-2.437582
H	3.756650	4.714900	-4.176337
H	2.070911	5.174945	-3.724913
C	3.597237	2.087046	-3.261953
H	3.925821	2.294974	-2.220616
H	3.242960	1.037160	-3.339772
H	4.466785	2.219251	-3.941101
O	0.870284	4.281954	-7.816418
H	-0.232548	1.473010	-6.904632
H	0.701047	2.241962	-9.460717
C	-0.647998	0.578175	-9.216029
H	-0.995960	0.502712	-10.267804
H	-1.484115	0.311643	-8.534740
O	1.625551	-0.014581	-8.814414
C	0.476230	-0.409644	-9.008576

H	0.827873	-2.385082	-8.759684
H	-0.753680	-1.980978	-9.337598
O	-1.055592	3.508394	-10.597900
H	-2.412587	2.919681	-7.823924
C	-3.277190	4.267691	-9.187573
H	-3.590827	3.969892	-10.176856
H	-4.123851	4.287685	-8.517376
H	-2.837508	5.250534	-9.271832
N	0.157210	-1.697753	-9.037652

1S2R

C	0.278980	-6.258851	-1.768261
H	0.090416	-7.327966	-1.721359
C	-0.509848	-5.437635	-2.579001
H	-1.314213	-5.870849	-3.168284
C	1.314968	-5.690782	-1.020526
H	1.935760	-6.317425	-0.384886
C	-0.276493	-4.066601	-2.645399
H	-0.897118	-3.463000	-3.293525
C	1.550787	-4.322824	-1.084749
H	2.352573	-3.884445	-0.495219
C	0.760527	-3.466568	-1.891309
C	1.103722	-2.057132	-1.900947
H	2.009511	-1.823971	-1.345376
C	0.436902	-0.948259	-2.403849
H	1.024381	-0.036783	-2.406945
C	-0.740438	-0.940537	-3.343483
H	-1.079402	0.085607	-3.506938
H	-0.473997	-1.374385	-4.317476
H	-1.581530	-1.505197	-2.932451
O	-0.593088	-0.362888	-0.811241
Fe	-0.229540	0.811612	0.358015
N	-1.761985	1.986548	-0.216491
N	1.042484	1.918912	-0.742317
N	1.350553	-0.187942	1.136971
N	-1.453153	-0.123613	1.676180
C	-3.063913	1.909798	0.200779
C	-3.879892	2.850795	-0.531823
C	-3.052902	3.487759	-1.404967
C	-1.731116	2.942419	-1.196601
C	0.697629	2.899142	-1.641825
C	1.876028	3.404484	-2.304616
C	2.938946	2.725480	-1.790866
C	2.408559	1.795420	-0.823310
C	2.674738	-0.043726	0.803601
C	3.474560	-1.029995	1.491006
C	2.614274	-1.784022	2.230767
C	1.291424	-1.250101	2.007021
C	-1.132541	-1.194932	2.469677
C	-2.307839	-1.672322	3.162211
C	-3.340450	-0.874265	2.778249

C	-2.799759	0.081159	1.838888
C	-3.549601	1.041746	1.172924
H	-4.609511	1.094759	1.402805
C	-0.591346	3.364244	-1.871563
H	-0.713469	4.142533	-2.618897
C	3.176703	0.892594	-0.093909
H	4.249387	0.898088	-0.263408
C	0.139678	-1.733060	2.615464
H	0.247627	-2.584862	3.280116
H	-3.296260	4.259947	-2.123823
H	-4.943777	2.988946	-0.386104
H	1.874580	4.183710	-3.056346
H	3.988271	2.828923	-2.036927
H	4.549621	-1.124131	1.401870
H	2.838882	-2.620949	2.879996
H	-2.320409	-2.506783	3.851936
H	-4.378071	-0.919051	3.084051
S	0.103110	2.365463	2.498037
C	1.560484	3.168618	2.458977
H	2.101184	3.285535	3.405903
H	2.333833	2.722630	1.821788
H	1.706360	4.994235	1.240025
C	1.518732	4.678786	2.160230
C	0.503309	5.346996	3.073136
N	-0.561456	5.933279	2.497240
C	-1.622159	6.607500	3.255081
C	-2.337296	5.669513	4.245140
N	-2.528659	4.402273	3.872570
C	-3.206241	3.442307	4.751875
C	-2.379269	3.090093	6.002600
N	-1.109735	2.745453	5.800533
C	-0.231588	2.402061	6.921469
C	-0.054655	3.581006	7.895160
N	0.119910	4.786058	7.355274
C	0.276727	5.967311	8.195726
C	-0.967898	6.244104	9.030240
N	-2.149821	6.043752	8.456855
N	2.852030	5.226798	2.339681
C	3.549390	5.749343	1.334682
C	4.970635	6.242806	1.680686
N	5.443363	5.522831	2.871654
C	5.173273	5.973257	4.116876
C	5.692991	5.133049	5.282496
N	5.502847	5.731566	6.606467
C	6.515183	6.178862	7.342195
C	6.172539	6.720549	8.728490
H	5.512962	5.992319	9.176090
H	5.690375	7.686149	8.689324
H	7.074666	6.760303	9.320931
O	7.683795	6.136933	6.967878
H	4.560295	5.783764	6.926434
H	6.751952	5.025804	5.101232

C	4.998378	3.775835	5.263823
H	5.704047	2.985296	5.598143
H	4.124186	3.784716	5.950271
H	4.640902	3.547854	4.236800
O	4.575513	7.039260	4.313962
C	6.479094	4.533091	2.508246
H	7.487171	4.918211	2.767681
H	6.293534	3.573140	3.032828
C	6.298379	4.398239	1.008118
H	7.225841	4.033928	0.516474
H	5.463858	3.709208	0.758355
C	5.981800	5.821042	0.610228
H	6.890014	6.460103	0.636193
H	5.538054	5.867634	-0.407122
H	4.927755	7.303423	1.870995
O	3.083868	5.882818	0.202256
H	3.216214	5.187681	3.266807
O	0.652142	5.315585	4.291541
C	-0.776736	6.087014	1.048024
H	0.186020	6.282768	0.532228
H	-1.254598	5.176868	0.628542
C	-1.698012	7.281930	0.994489
H	-1.129708	8.231591	1.085174
H	-2.278221	7.298856	0.047746
C	-2.583213	7.077672	2.158138
H	-3.354994	6.307838	1.944555
H	-3.089747	8.022533	2.447534
H	-1.208867	7.397182	3.862799
O	-2.713683	6.094926	5.337147
H	-2.223097	4.053732	2.989569
H	-4.093684	3.915848	5.144412
C	-3.576007	2.180823	3.981558
H	-4.098155	2.452239	3.039360
H	-2.659494	1.606469	3.726488
H	-4.236018	1.537856	4.602967
O	-2.877516	3.181769	7.139481
H	-0.704041	2.706108	4.890629
H	-0.712801	1.627854	7.500467
C	1.126462	1.915959	6.406794
H	1.533419	2.693682	5.727496
H	1.804360	1.808191	7.279844
C	1.118138	0.582142	5.646583
H	0.319873	0.602462	4.873598
C	0.762480	-0.570369	6.576999
H	0.753801	-1.525777	6.009248
H	1.502960	-0.632387	7.402221
H	-0.245770	-0.409876	7.015473
C	2.465578	0.348367	4.977635
H	2.455291	-0.621879	4.435326
H	2.682742	1.172641	4.265427
H	3.271477	0.318605	5.742144
O	-0.091253	3.403842	9.115720

H	0.151111	4.932999	6.369686
H	1.049883	5.760413	8.919990
C	0.651760	7.189711	7.355833
H	0.583488	8.099528	7.988740
H	-0.061120	7.279939	6.508515
O	2.811127	6.198058	7.167596
C	2.052920	7.094574	6.798689
H	3.261000	7.904049	5.393964
H	1.817734	8.798223	5.734598
O	-0.856378	6.612618	10.193854
H	-2.244784	5.733865	7.513726
C	-3.384697	6.272353	9.197423
H	-3.327839	7.252687	9.646249
H	-4.242746	6.209492	8.544582
H	-3.466580	5.555130	10.000720
N	2.406203	8.007293	5.902040

Data S2B. Cartesian coordinates of the 1R2S stationary points.

Reactant, model A

C	-3.97104521	4.01514205	5.23827324
H	-4.66333693	4.84055684	5.09558777
C	-2.60992920	4.26096393	5.43383012
H	-2.23936265	5.28286158	5.45291700
C	-4.43714480	2.69745044	5.25207168
H	-5.49659867	2.49247915	5.11999258
C	-1.71931007	3.20318494	5.62278804
H	-0.67141382	3.41361380	5.80827576
C	-3.54630048	1.64270890	5.44570163
H	-3.91768082	0.62018616	5.46426100
C	-2.16572313	1.86887361	5.61182974
C	-1.27087902	0.71008608	5.80076390
H	-1.72775559	-0.12388301	6.33451990
C	-0.01148917	0.53354668	5.36232370
H	0.45674133	-0.41754426	5.61806631
C	0.83531625	1.43914800	4.51559033
H	1.25970188	0.87726839	3.67397647
H	0.27447964	2.28212265	4.10561910
H	1.68280133	1.83959144	5.09136543
O	1.61364349	-0.08522826	1.30716467
Fe	0.66534868	-0.00132116	-0.00715916
N	-0.86665047	-1.01293798	0.81407256
N	1.36425723	-1.71788292	-0.80194871
N	2.01268826	1.04040472	-1.10524240
N	-0.19476945	1.74259087	0.53990053
C	0.91513962	-2.98812252	-0.53377700
C	1.76398057	-3.96350265	-1.17350703
C	2.73626925	-3.26866022	-1.82556721
C	2.47980610	-1.86980017	-1.58248237

C	3.03611963	0.51911644	-1.86327332
C	3.87422797	1.58017575	-2.36618594
C	3.36522339	2.74654254	-1.88444018
C	2.21055144	2.40486436	-1.09172027
C	0.29998764	3.00524137	0.34156787
C	-0.52552112	3.97884113	1.01551559
C	-1.52807094	3.28981279	1.62528374
C	-1.30948354	1.89426501	1.32734683
C	-1.89521785	-0.48987187	1.55133920
C	-2.73293843	-1.54658722	2.06458750
C	-2.19152044	-2.71833809	1.63328610
C	-1.02624490	-2.37293749	0.85533821
C	-0.19851444	-3.29897078	0.23253312
H	-0.44316697	-4.34963090	0.35550173
C	3.25445669	-0.83168767	-2.09110542
H	4.11094359	-1.10229033	-2.70121212
C	1.42518612	3.31983407	-0.41136695
H	1.70197081	4.36713958	-0.48238955
C	-2.11205274	0.86242837	1.78963917
H	-2.95906853	1.13180591	2.41233293
H	3.55718742	-3.65085921	-2.41891358
H	1.62034445	-5.03516836	-1.11973717
H	3.72698656	3.75494010	-2.03984391
H	4.74230279	1.43230082	-2.99599064
H	-0.34720897	5.04661221	1.00788301
H	-2.33829881	3.66781423	2.23514443
H	-3.61375069	-1.39173907	2.67422512
H	-2.53530628	-3.72902681	1.81293768
S	-0.65965853	0.49206607	-2.21722156
C	-0.07702581	-0.62344295	-3.52654430
H	-0.63361447	-0.42259363	-4.44670163
H	0.98538562	-0.42602066	-3.71610964

H	-0.18486878	-1.67162782	-3.23603766

Reactant, model B

C	-4.77517268	1.99048726	4.93904421
H	-5.60850911	2.66317954	4.75535819
C	-3.56375443	2.48450156	5.42947023
H	-3.45549403	3.54538364	5.64137314
C	-4.91397561	0.61719055	4.71735027
H	-5.85941725	0.21552989	4.36125712
C	-2.49439306	1.62151655	5.67602229
H	-1.57488609	2.01703244	6.09398042
C	-3.84590824	-0.24350634	4.96871606
H	-3.96522476	-1.31266578	4.80636768
C	-2.60532557	0.24041365	5.42950099
C	-1.50925530	-0.72023204	5.66361441
H	-1.84257156	-1.70412886	5.99456519
C	-0.18723252	-0.55933287	5.47493959
H	0.43923567	-1.41986761	5.71149410
C	0.55776469	0.62548007	4.93132809
H	1.23069064	0.31017883	4.12412881
H	-0.10415581	1.39492027	4.52813982
H	1.18529585	1.08728232	5.70745635
O	1.84437762	0.13718972	1.52197571
Fe	1.13903309	0.48864336	0.10731461
N	-0.46266093	-0.68834966	0.43839027
N	2.02203773	-1.02035871	-0.89222519
N	2.59025783	1.73862838	-0.51408072
N	0.10884682	2.06108658	0.83149860
C	1.58423494	-2.31582648	-0.99426607
C	2.56539216	-3.12956516	-1.66890982
C	3.61176964	-2.31124368	-1.96760914
C	3.26402273	-1.00019573	-1.47931669

C	3.74437367	1.39885918	-1.16880225
C	4.58003742	2.56025260	-1.35909144
C	3.91378150	3.61198213	-0.81049534
C	2.67832102	3.08602425	-0.28179912
C	0.54070982	3.36219272	0.91847429
C	-0.39568521	4.15462509	1.67737730
C	-1.39143190	3.31384944	2.07071004
C	-1.06738223	2.01257985	1.53982973
C	-1.56272420	-0.37616138	1.20221192
C	-2.39933780	-1.53830917	1.37786120
C	-1.79838036	-2.56111969	0.71096927
C	-0.57939180	-2.03090359	0.15262422
C	0.36110132	-2.78803760	-0.52749106
H	0.12476067	-3.83208824	-0.70234577
C	4.06875421	0.12234365	-1.61039383
H	5.02500256	-0.00499984	-2.10837115
C	1.72464288	3.84837449	0.38085864
H	1.93309420	4.90609028	0.50890653
C	-1.84311580	0.87704245	1.72861319
H	-2.73641440	0.97596983	2.33718052
H	4.53794147	-2.55507899	-2.47190215
H	2.45463245	-4.18724861	-1.87086514
H	4.21468235	4.65027120	-0.75832768
H	5.54337599	2.55315201	-1.85235517
H	-0.28413857	5.21191106	1.88058474
H	-2.26575021	3.52865503	2.67110998
H	-3.32048810	-1.54695866	1.94587116
H	-2.12650473	-3.58593141	0.59964000
S	-0.19520091	1.13859982	-2.23835199
C	-0.35492563	-0.36644629	-3.25954576
H	-0.04320548	-0.06765069	-4.26912653
H	0.34567800	-1.12862639	-2.91583771

H	-1.98848673	-1.47684152	-2.40608958
C	-1.76147299	-1.00045189	-3.35677678
C	-2.82084585	0.05217352	-3.72791930
N	-3.81038231	0.31594169	-2.83857244
C	-4.87026078	1.28890334	-3.17606892
C	-4.37378068	2.70985406	-3.47339384
N	-3.32356850	3.14909735	-2.73568518
C	-2.81439646	4.51655651	-2.84977210
C	-2.14043969	4.76974086	-4.21658248
N	-1.22218466	3.84107884	-4.60863073
C	-0.57542373	3.93488668	-5.91541779
C	-1.57400880	3.84345263	-7.08972499
N	-2.57219527	2.91392805	-7.01024796
C	-3.52990734	2.84328778	-8.11172311
C	-4.37713776	4.12836461	-8.29589854
N	-4.77784237	4.73917392	-7.15198259
N	-1.70721985	-2.07122587	-4.34368909
C	-1.88662197	-3.37320892	-3.97433143
C	-1.83781028	-4.44229291	-5.09076751
N	-1.07663528	-4.03987066	-6.28992051
C	-1.72349359	-3.35572015	-7.28028876
C	-0.91347855	-2.92024153	-8.51552380
N	-1.77720394	-2.34488848	-9.51650526
C	-2.01333668	-2.99712786	-10.69263493
C	-2.92625154	-2.25942289	-11.65965224
H	-3.22143694	-1.26945266	-11.30161295
H	-3.82312040	-2.86406821	-11.83153863
H	-2.41353524	-2.15946871	-12.62136869
O	-1.52672462	-4.09456494	-10.96173680
H	-2.22012470	-1.44442798	-9.31175984
H	-0.44019874	-3.79615092	-8.97142893
C	0.14858260	-1.87069572	-8.10886147

H	0.77371328	-1.63942172	-8.97583319
H	-0.34749202	-0.94403846	-7.79894743
H	0.79519607	-2.20661820	-7.29275546
O	-2.89529347	-3.00464551	-7.09969971
C	0.16681148	-4.82466389	-6.44436245
H	0.05082335	-5.56201981	-7.24928449
H	1.01704651	-4.18470052	-6.69231920
C	0.32448739	-5.51193354	-5.08076257
H	0.89590685	-6.44156304	-5.15432460
H	0.85048117	-4.84646969	-4.38492171
C	-1.12588910	-5.72185762	-4.61902219
H	-1.56303719	-6.58948200	-5.12555797
H	-1.23408744	-5.85737682	-3.54217611
H	-2.87791519	-4.62789941	-5.37712846
O	-2.06985296	-3.71540827	-2.80572848
H	-1.76419524	-1.81524719	-5.32136075
O	-2.72185292	0.66769515	-4.80066946
C	-4.17299053	-0.50409694	-1.65621229
H	-3.97176322	-1.56255768	-1.83271232
H	-3.58923920	-0.18681997	-0.78573067
C	-5.66789515	-0.21349473	-1.46602568
H	-6.26433925	-0.88956327	-2.08979928
H	-5.98417724	-0.34877177	-0.42765221
C	-5.81403516	1.23246846	-1.95868308
H	-5.46873494	1.93449765	-1.19053507
H	-6.83571309	1.50686372	-2.23143598
H	-5.38725287	0.96663559	-4.08649921
O	-4.97950368	3.41122430	-4.27988137
H	-2.77888908	2.47321568	-2.20905039
H	-3.67417881	5.19125586	-2.84418262
C	-1.87765486	4.84850471	-1.68816995
H	-2.39688370	4.74737685	-0.72956573

H	-1.00041463	4.19374017	-1.67862230
H	-1.53042309	5.88154095	-1.78718182
O	-2.37297905	5.79160254	-4.85078921
H	-1.18604983	2.95671184	-4.11440225
H	-0.16408642	4.94187232	-6.01215079
C	0.53859210	2.88998461	-6.07915962
H	0.09762877	1.88652162	-5.98418652
H	0.89689647	2.97023135	-7.11371886
C	1.73685687	3.01950648	-5.11582886
H	1.36551748	2.91537762	-4.08666561
C	2.44303358	4.37852485	-5.22965739
H	3.30514866	4.42094769	-4.55308932
H	2.80892192	4.54879882	-6.25063479
H	1.78007325	5.21041660	-4.96996763
C	2.72912562	1.87344336	-5.36627444
H	3.55834375	1.90957592	-4.64988661
H	2.24545713	0.89369996	-5.26678691
H	3.15410808	1.93419476	-6.37677225
O	-1.40698339	4.54269669	-8.08097275
H	-2.68440236	2.32615553	-6.19152519
H	-2.98276140	2.72191166	-9.04849375
C	-4.50425945	1.67723660	-7.94149100
H	-5.28070547	1.79310564	-8.70885529
H	-5.00798231	1.74046854	-6.96869660
O	-2.88179227	0.12667955	-8.84647509
C	-3.88422673	0.30154079	-8.14186917
H	-4.10314242	-1.63905018	-7.54735567
H	-5.26985170	-0.53171496	-6.87962272
O	-4.74692785	4.45118866	-9.42047376
H	-4.39033826	4.43757931	-6.26681937
C	-5.53363678	5.97707956	-7.16004535
H	-5.80592199	6.19375408	-8.19413657

H	-6.44410358	5.87886126	-6.55664759
H	-4.93264963	6.80211444	-6.75981234
N	-4.55147993	-0.72245774	-7.56173945

Transition State, model A

C	-0.65958926	4.41724533	6.14176605
H	-0.43069756	5.25581653	6.79382453
C	-0.09663947	3.16096295	6.38401235
H	0.57076537	3.02041656	7.23048866
C	-1.52410211	4.58179553	5.05528263
H	-1.97132911	5.55239654	4.85621929
C	-0.38456013	2.08027141	5.55531119
H	0.05473131	1.11790706	5.78078322
C	-1.81538340	3.50459675	4.22663950
H	-2.48357511	3.64047859	3.37971635
C	-1.25054236	2.22347924	4.44463339
C	-1.63188172	1.16302517	3.53200716
H	-2.40789169	1.45238657	2.82722978
C	-1.12035929	-0.11103564	3.33622004
H	-1.72024982	-0.74062599	2.68794755
C	-0.15463608	-0.85488776	4.22087481
H	0.12174007	-1.80249878	3.75144142
H	0.76442887	-0.28404120	4.37758536
H	-0.60088137	-1.07548554	5.20076707
O	0.19089963	0.22971609	1.86980219
Fe	-0.02657650	0.12661276	0.18516843
S	-0.02913068	0.38501340	-2.31311106
C	1.40050841	-0.48345239	-3.02692332
H	1.37333995	-0.36788185	-4.11481722
H	2.32728296	-0.03028627	-2.65610940
H	1.40102606	-1.54345262	-2.76030591
N	1.32513014	-1.37580977	0.09275941

N	1.48795745	1.46694393	-0.01924704
N	-1.37078035	1.62289383	0.06508211
N	-1.52974513	-1.20760924	0.17993404
C	1.05743418	-2.71570432	0.20936757
C	2.28852115	-3.47227309	0.22486421
C	3.30218093	-2.56992720	0.12159537
C	2.68859962	-1.26374404	0.04859303
C	2.83297520	1.20139915	-0.06221643
C	3.58522335	2.43605559	-0.08372615
C	2.67832502	3.44781692	-0.02635585
C	1.37007718	2.83484840	0.01838248
C	-1.09632254	2.96602320	0.07779740
C	-2.32418531	3.72586728	0.04784736
C	-3.34411416	2.82437878	0.00076954
C	-2.73861113	1.51394726	0.01772278
C	-2.87498334	-0.94950655	0.10381872
C	-3.62574268	-2.18182665	0.14101010
C	-2.71597758	-3.19002708	0.23966835
C	-1.41090213	-2.57200214	0.25055870
C	-0.21151076	-3.27593185	0.28540611
H	-0.27366413	-4.35797610	0.35534432
C	3.39611565	-0.06820295	-0.04611759
H	4.47981957	-0.13303452	-0.07514923
C	0.17398675	3.53344917	0.07996675
H	0.23194137	4.61750762	0.10086906
C	-3.44363288	0.31703601	0.01748756
H	-4.52681594	0.37506892	-0.03346492
H	4.36908097	-2.75293949	0.09939979
H	2.34996100	-4.55036102	0.30388343
H	2.85900193	4.51519186	-0.01434744
H	4.66525104	2.49969312	-0.12512449
H	-2.37958284	4.80713671	0.04575120

H	-4.40953466	3.01206750	-0.04241682
H	-4.70532391	-2.24888811	0.09498611
H	-2.89417398	-4.25687757	0.28772840

Transition State, model B

C	-1.40725368	3.64104807	6.72345023
H	-1.37004363	4.44501362	7.45310168
C	-0.58893470	2.51655263	6.87504718
H	0.08102865	2.44514793	7.72735648
C	-2.27923221	3.71997153	5.63300773
H	-2.92267800	4.58680977	5.51090266
C	-0.62873936	1.48203755	5.94698026
H	-0.00114182	0.61475164	6.10201078
C	-2.32638938	2.68650914	4.70596727
H	-3.00346998	2.74941311	3.85803073
C	-1.49526806	1.54441005	4.82813132
C	-1.62494789	0.52176659	3.81349304
H	-2.45778374	0.67913262	3.13235456
C	-0.83705751	-0.57616995	3.51537146
H	-1.25406128	-1.24925224	2.77337820
C	0.34333874	-1.10894476	4.27300382
H	0.86392285	-1.85688823	3.67015292
H	1.05710831	-0.31871364	4.51890256
H	0.02326561	-1.59144654	5.20786356
O	0.30548894	0.32218577	1.97763108
Fe	0.14242552	0.44959474	0.33301015
N	-0.77959880	-1.31676610	0.07412030
N	1.93519729	-0.45932206	0.11363273
N	1.06786004	2.24753031	0.38690133
N	-1.64220459	1.37385638	0.37197532
C	2.16848289	-1.80897947	0.05328383
C	3.58903341	-2.06969262	0.03635106

C	4.20892223	-0.85962809	0.08831717
C	3.16800608	0.13990088	0.14460817
C	2.41744844	2.47757747	0.35613862
C	2.68718887	3.89009871	0.50591446
C	1.48308991	4.50543387	0.64366739
C	0.47806757	3.46909141	0.57199785
C	-1.86751408	2.71293301	0.57354389
C	-3.28449158	2.97347314	0.64717742
C	-3.91450505	1.77624071	0.48234157
C	-2.88153318	0.78336674	0.31597314
C	-2.13386931	-1.54945541	0.04479864
C	-2.39320526	-2.96126698	-0.10235663
C	-1.18236678	-3.58100503	-0.15949111
C	-0.18142999	-2.55008497	-0.03826830
C	1.18623840	-2.78842368	-0.02965220
H	1.51015373	-3.82024263	-0.11798950
C	3.39968197	1.50517125	0.23631344
H	4.43322489	1.83650826	0.23899859
C	-0.88754104	3.69267486	0.67207884
H	-1.21767702	4.71557151	0.82315642
C	-3.11817194	-0.57595631	0.15751430
H	-4.15276236	-0.90497013	0.13608369
H	5.26957197	-0.64343569	0.09249680
H	4.03395036	-3.05554055	-0.01112645
H	1.27343920	5.55876386	0.77806884
H	3.67567917	4.33099790	0.50850928
H	-3.72338643	3.95255007	0.79179935
H	-4.97704973	1.56872357	0.47158248
H	-3.37975378	-3.40377506	-0.15839945
H	-0.96574501	-4.63213723	-0.29381172
S	0.15741161	0.82605305	-2.21550491
C	0.96870219	-0.60215809	-3.03808125

H	1.69278471	-0.18950683	-3.74981955
H	1.52419263	-1.18526380	-2.30427125
H	-0.53387329	-2.14313529	-3.07744711
C	0.03135153	-1.56363880	-3.80297436
C	-0.89865665	-0.79631915	-4.75731663
N	-2.23631958	-0.85332822	-4.53567680
C	-3.16836567	-0.15690448	-5.44372809
C	-2.96235353	1.36053597	-5.53863729
N	-2.59879475	1.99800061	-4.39728050
C	-2.46297297	3.45460387	-4.34539049
C	-1.26291052	3.95780060	-5.17884780
N	-0.07399132	3.33019584	-4.96711512
C	1.10374521	3.68199427	-5.75652741
C	0.91063314	3.41435157	-7.26450806
N	0.29328447	2.25338584	-7.63460202
C	0.10341969	2.00795954	-9.06215089
C	-0.81991518	3.03702252	-9.76325249
N	-1.89744971	3.44620526	-9.04656607
N	0.85743689	-2.51989230	-4.53274693
C	0.84846037	-3.84578925	-4.21617560
C	1.75119998	-4.78499909	-5.05183085
N	2.90055208	-4.11919936	-5.69533833
C	2.72914080	-3.55498606	-6.92807062
C	3.92292785	-2.82078786	-7.56671001
N	3.61232877	-2.41816474	-8.91620421
C	4.22646591	-3.01197707	-9.98076183
C	3.81036552	-2.47153020	-11.34019207
H	3.11069493	-1.63407395	-11.27373203
H	3.35193759	-3.28120489	-11.91795052
H	4.70650940	-2.15370183	-11.88225950
O	5.05788188	-3.91143403	-9.86231955
H	2.90183713	-1.69300190	-9.05288141

H	4.78101738	-3.49822184	-7.62807557
C	4.27437089	-1.56234877	-6.73760432
H	5.17533903	-1.10067923	-7.15125475
H	3.46136733	-0.83131505	-6.81171425
H	4.44817910	-1.77923214	-5.67943538
O	1.60104570	-3.54658375	-7.43337448
C	4.18724482	-4.53229391	-5.09751172
H	4.71908573	-5.21093528	-5.77707463
H	4.83452640	-3.67347483	-4.90323422
C	3.76488072	-5.25409662	-3.80976303
H	4.50503471	-5.99485895	-3.49382209
H	3.64545931	-4.52792185	-2.99620094
C	2.40394766	-5.86714760	-4.17483158
H	2.54522105	-6.77924499	-4.76546584
H	1.77819173	-6.10333449	-3.31327944
H	1.10876638	-5.21165179	-5.82834472
O	0.16561778	-4.31176942	-3.30342551
H	1.28455395	-2.21050552	-5.39599987
O	-0.41644578	-0.11326687	-5.67364050
C	-2.94256053	-1.80997948	-3.64956957
H	-2.42224066	-2.76904728	-3.60849447
H	-2.99789623	-1.40688468	-2.63264368
C	-4.33442635	-1.92269457	-4.28653356
H	-4.32646021	-2.68520326	-5.07429983
H	-5.10047130	-2.20279257	-3.55724318
C	-4.55912859	-0.53182267	-4.89468199
H	-4.85290890	0.18218691	-4.11622894
H	-5.31931550	-0.50769441	-5.67901146
H	-3.03717237	-0.53101469	-6.46504363
O	-3.22189068	1.94283543	-6.58915410
H	-2.23319496	1.45016272	-3.62297305
H	-3.33402013	3.88439877	-4.84587719

C	-2.39066472	3.94340172	-2.89899732
H	-3.28976288	3.65055452	-2.34664012
H	-1.51713338	3.53626292	-2.38030294
H	-2.31544701	5.03531005	-2.88887005
O	-1.38943172	4.90961071	-5.94172072
H	-0.03870902	2.51002822	-4.37126526
H	1.23449257	4.76546528	-5.70836661
C	2.36980665	2.97206669	-5.25491316
H	2.20274250	1.88565482	-5.29084932
H	3.15991563	3.18670846	-5.98641394
C	2.86571868	3.37025993	-3.84930840
H	2.08223928	3.12130298	-3.12112460
C	3.17080478	4.87107488	-3.73458004
H	3.54824071	5.11128046	-2.73310664
H	3.93351330	5.17650959	-4.46292622
H	2.28173340	5.48708114	-3.90529063
C	4.10893721	2.53962947	-3.49490603
H	4.46371469	2.77410263	-2.48434987
H	3.89598461	1.46428712	-3.53150129
H	4.93076762	2.74311903	-4.19419428
O	1.37817597	4.19693759	-8.08271458
H	-0.07078758	1.60523006	-6.94438586
H	1.06715821	2.09433136	-9.56762915
C	-0.48234545	0.62051370	-9.32607381
H	-0.75074423	0.58225921	-10.38987144
H	-1.41190708	0.48573729	-8.75903036
O	1.70705408	-0.39603925	-9.15862610
C	0.47978472	-0.53109385	-9.07198385
H	0.49180562	-2.49393636	-8.51479980
H	-1.08605532	-1.76604366	-8.60071809
O	-0.62153251	3.32265826	-10.94040918
H	-1.95698574	3.20999304	-8.06387725

C	-2.82382509	4.44367576	-9.54635000
H	-2.58218587	4.62696144	-10.59446724
H	-3.85688051	4.08451659	-9.46708992
H	-2.73194301	5.37922088	-8.98175075
N	-0.10409055	-1.72726606	-8.82893142
